# Leptin signaling and its central role in energy homeostasis

**DOI:** 10.3389/fnins.2023.1238528

**Published:** 2023-10-31

**Authors:** Zhaoxun Liu, Tao Xiao, Hailan Liu

**Affiliations:** ^1^Nursing Department, The Third Xiangya Hospital, Central South University, Changsha, Hunan, China; ^2^Department of Emergency, The Third Xiangya Hospital, Central South University, Changsha, Hunan, China; ^3^USDA/ARS Children’s Nutrition Research Center, Department of Pediatrics, Baylor College of Medicine, Houston, TX, United States

**Keywords:** leptin, central nervous system, energy balance, food intake, leptin signaling

## Abstract

Leptin plays a critical role in regulating appetite, energy expenditure and body weight, making it a key factor in maintaining a healthy balance. Despite numerous efforts to develop therapeutic interventions targeting leptin signaling, their effectiveness has been limited, underscoring the importance of gaining a better understanding of the mechanisms through which leptin exerts its functions. While the hypothalamus is widely recognized as the primary site responsible for the appetite-suppressing and weight-reducing effects of leptin, other brain regions have also been increasingly investigated for their involvement in mediating leptin’s action. In this review, we summarize leptin signaling pathways and the neural networks that mediate the effects of leptin, with a specific emphasis on energy homeostasis.

## Introduction

The global prevalence of obesity has reached alarming levels due to the widespread availability of palatable calorie-dense food and sedentary lifestyles (Lister et al., 2023). Obesity constitutes a major risk factor for cardiovascular diseases, type 2 diabetes mellitus, and neurodegenerative disorders, which not only diminish healthy lifespans but also impose a significant economic burden on the world economy (Kopelman, 2000). The central nervous system (CNS), particularly the hypothalamus, plays a pivotal role in controlling food intake, energy expenditure and body weight (Myers and Olson, 2012; Seong et al., 2019). The hypothalamus is composed of various nuclei, including the arcuate (ARH), ventromedial (VMH), dorsomedial (DMH), paraventricular (PVH) nucleus of the hypothalamus, and lateral hypothalamic area (LH; Dietrich and Horvath, 2013). In recent years, accumulating evidence has also emphasized the involvement of neurons beyond the hypothalamus in the control of energy homeostasis (Waterson and Horvath, 2015).

Peripheral hormones and nutrients, such as leptin, can act on the CNS to finely regulate whole-body energy balance (Xu et al., 2011). Notably, leptin is one of the most extensively studied and well-defined hormones that play essential roles in suppressing appetite and enhancing energy expenditure (Friedman and Halaas, 1998). Leptin (ob/ob) or leptin receptor (db/db) deficient mice and humans developed pronounced hyperphagia and obesity, suggesting leptin is a key player in the control of feeding and energy balance (Zhang et al., 1994; Montague et al., 1997; Cohen et al., 2001). Remarkably, multiple clinical studies have conclusively demonstrated the efficacy of leptin in the amelioration of obesity linked to congenital leptin insufficiency (Kilpeläinen et al., 2016; Dallner et al., 2019; Lawler et al., 2020; Hanssen et al., 2023; Saeed et al., 2023). Intracerebroventricular (ICV) administration of leptin into ob/ob mice or re-expression of leptin receptor in the CNS of db/db mice can fully reverse the corresponding metabolic disorders (de Luca et al., 2005), highlighting the involvement of the CNS in mediating the effects of leptin. This review aims to enhance our comprehension of leptin signaling and the neural populations that express LepRb as well as their impact on energy homeostasis, which may pave the way for the development of novel therapeutic strategies to address metabolic disorders stemming from dysregulated energy balance.

## Leptin signaling pathways

Leptin was initially cloned in 1994, and this groundbreaking discovery has had a profound impact on our understanding of the mechanisms that underlie obesity and metabolic disorders (Zhang et al., 1994). Leptin exerts its actions by binding to leptin receptors in various tissues, especially in the CNS. Rodents have six identified leptin receptor isoforms (LepRa-f), which fall into three groups: secreted, short, and long isoforms. Among these isoforms, the secreted isoform, LepRe, contains only the extracellular domain responsible for binding to leptin. It lacks the transmembrane and cytoplasmic domains found in other isoforms. On the other hand, the short isoforms, namely LepRa, LepRc, LepRd, and LepRf, along with the long leptin receptor isoform LepRb, comprise both the intracellular, transmembrane, and extracellular domains (Tartaglia et al., 1995). Particularly, LepRb plays essential roles in transmitting leptin signals into the cells and initiating the biological responses associated with leptin’s weight-regulating effects (Tartaglia et al., 1995; Banks et al., 2000). Upon binding to LepRb, leptin triggers multiple signaling cascades, including JAK2-STAT3, AMPK, mTOR-S6K, PI3K-AKT-FOXO1, PI3K-PDE3B-cAMP and SHP2-ERK signaling pathways (Hübschle et al., 2001; Zhao et al., 2002; Ren et al., 2005; Xu et al., 2005; Kitamura et al., 2006; Harlan et al., 2013).

### JAK2-STAT3 pathway

Leptin binding with LepRb triggers the dimerization of LepRb, leading to the autophosphorylation and activation of JAK2 (Janus tyrosine kinase 2). Subsequently, activated JAK2 phosphorylates the tyrosine residues of LepRb at positions 985, 1,077, and 1,138. Among these, phosphorylated Tyr1138 plays a crucial role as it strongly recruits and phosphorylates STAT3 (signal transducers and activators of transcription; Kisseleva et al., 2002). Once phosphorylated, STAT3 forms homodimers and translocates from the cytoplasm to the nucleus, where it binds to the DNA promoter, up-regulating the expression of the anorexigenic gene *Pomc* and down-regulating the expression of the orexigenic genes *Npy/Agrp* (Hübschle et al., 2001). Notably, mice with LepRb Tyr1138 replaced by serine (s/s mice) exhibit decreased *Pomc* expression, leading to severe hyperphagia and obesity. Moreover, specific deletion of STAT3 in neurons also results in profound obesity in mice (Bates et al., 2003). Conversely, AMP-dependent protein kinase A (PKA) deficient mice show enhanced sensitivity to leptin due to an increase in the duration of hypothalamic JAK2-STAT3 signaling (Yang and McKnight, 2015). Recent research has unveiled the role of histone deacetylase 5 (HDAC5) in mediating leptin signaling in the hypothalamus. HDAC5 deacetylates STAT3 at Lys685, contributing to increased STAT3 activation. Mice with HDAC5 knocked down in the medial basal hypothalamus (MBH) show an obese phenotype characterized by hyperphagia and diminished hypothalamic leptin signaling. Intriguingly, overexpression of HDAC5 in the hypothalamus protects mice from diet-induced obesity by enhancing leptin-stimulated STAT3 phosphorylation, which subsequently elevates *Pomc* expression and inhibits food intake (Kabra et al., 2016).

Numerous molecules have been discovered to play essential roles in regulating the JAK2-STAT3 pathway, which influences various aspects of energy balance and sensitivity to leptin. Understanding the functions of these molecules is crucial for identifying potential therapeutic targets for obesity and related metabolic disorders. One such molecule is SH2B1, an adaptor protein containing the SH2 domain. SH2B1 interacts with JAK2 and modulates leptin sensitivity and energy balance. Mice lacking SH2B1 display severe obesity, elevated expression of AgRP/NPY, and impaired activation of JAK2 and STAT3. Conversely, overexpressing SH2B1 enhances leptin sensitivity, suggesting its potential as a therapeutic target for obesity (Ren et al., 2005). Rho-kinase 1 (ROCK1) has also been identified as a positive regulator of leptin signaling. Leptin promotes the physical interaction between JAK2 and ROCK1, leading to increased JAK2 phosphorylation and downstream STAT3 activation. Deletion of ROCK1 in the ARH results in impaired leptin sensitivity, increased food intake, and severe obesity, highlighting ROCK1 as a critical modulator of leptin’s action on energy homeostasis (Huang et al., 2012). Steroid receptor coactivator-1 (SRC-1) interacts with phosphorylated STAT3 to enhance Pomc transcription, which influences energy balance. Deletion of SRC-1 in mice impairs hypothalamic leptin signaling, leading to hyperphagia and obesity induced by high-fat diet (Yang et al., 2019). Another molecule, growth factor receptor binding protein 10 (Grb10), interacts with LepRb and enhances the leptin-stimulated JAK2-STAT3 pathway (Liu et al., 2023). This interaction may have implications for targeting Grb10 in the treatment of obesity. On the other hand, the expression of suppressor of cytokine signaling 3 (SOCS3) is induced by STAT3 phosphorylation, which, in turn, diminishes leptin signaling by binding to JAK2. Mice lacking SOCS3 in the brain or POMC neurons display improved leptin sensitivity and resistance to diet-induced obesity (Howard et al., 2004; Kievit et al., 2006). Protein tyrosine phosphatase (PTP)1B and TCPTP counteract leptin-induced signaling by dephosphorylating tyrosine-phosphorylated substrates. PTP1B dephosphorylates JAK2, and PTP1B-deficient mice exhibit suppressed appetite, increased ambulatory activity, improved leptin sensitivity, and resistance to diet-induced obesity (Zabolotny et al., 2002). Similarly, TCPTP dephosphorylates STAT3, and mice lacking TCPTP show reduced food intake, increased energy expenditure, lower body weight, and improved leptin sensitivity compared with control mice (Loh et al., 2022).

### AMPK pathway

Adenosine monophosphate-activated protein kinase (AMPK) is a highly conserved intracellular energy sensor that responds to peripheral and central nutrients and hormones to regulate energy homeostasis. It exists as a heterotrimeric kinase composed of a catalytic subunit α and regulatory subunits β and γ (Andersson et al., 2004). AMPK is activated when the intracellular AMP/ATP ratio increases, such as during fasting, stress, or hypoxia. It can also be activated by upstream kinases, such as liver kinase B1 (LKB1), a tumor-suppressor gene product linked to Peutz-Jeghers syndrome, and Ca2+/calmodulin-dependent protein kinase kinase-β (CaMKKβ), which phosphorylate threonine 172 on the catalytic α subunit (Hawley et al., 2003). Previous studies have demonstrated that leptin activation of AMPK promotes lipolysis and fatty acid oxidation in skeletal muscle. Additionally, leptin administration activates AMPK to suppress hepatic glucose production in the liver and counteracts lipolysis in adipose tissue. Recently, it was discovered that hypothalamic AMPK activity is attenuated by high glucose levels, feeding, and leptin injection. Conversely, AMPK in the hypothalamus is activated by the orexigenic hormone ghrelin and energy deprivation conditions, such as fasting. Leptin-induced inactivation of AMPK leads to decreased acetyl-CoA carboxylase (ACC) and increased malonyl-CoA levels in hypothalamic neurons (Minokoshi et al., 2002). This increase in hypothalamic malonyl-CoA results in elevated intracellular long-chain fatty acetyl-CoA (LCFA-CoA) levels, which are believed to induce anorexia and suppress hepatic glucose production (Minokoshi et al., 2002). Activation of AMPK in the mediobasal hypothalamus stimulates food intake, while inhibition of AMPK reduces feeding. More specifically, mice lacking AMPK in POMC neurons developed obesity due to increased food intake and decreased energy expenditure. In contrast, mice lacking AMPK in AgRP neurons showed an age-dependent lean phenotype due to increased sensitivity to MT-II, a melanocortin agonist (Claret et al., 2007). These studies highlight the divergent effects of AMPK in POMC and AgRP neurons on energy homeostasis. Additionally, studies have suggested that phosphorylation of AMPK α2 subunit at serine 491 inhibits its activity by suppressing threonine 172 phosphorylation. A study by Dagon et al. showed that p70 S6K can form a complex with the AMPK α catalytic subunit and directly phosphorylate AMPK at serine 491 to suppress its activity (Dagon et al., 2012). These results imply that hypothalamic AMPK is a critical regulator of whole-body energy homeostasis, and serine 491 integrates multiple signaling pathways in the hypothalamus to mediate leptin’s actions.

### PI3K-AKT-mTOR pathway

Phosphatidylinositol 3-OH kinase (PI3K) is an enzyme that accelerates the production of phosphatidylinositol-3, 4, 5-trisphosphate (PIP3) from phosphatidylinositol-4, 5-bisphosphate (PIP2). Leptin stimulates the insulin receptor substrate (IRS)-PI3K signaling in the hypothalamus (Xu et al., 2005). Mice with an IRS null mutation exhibited increased food intake, decreased energy expenditure, and reduced responsiveness to exogenous leptin (Withers et al., 1998). Furthermore, intracerebroventricular (ICV) administration of a PI3K inhibitor significantly blocked the anorexigenic effects of leptin. Similarly, mice with a PI3K null mutation were obese and unresponsive to leptin (Hill et al., 2008). Protein kinase B (PKB), also known as AKT, is a downstream target of PI3K. Leptin treatment increases the phosphorylation of AKT at T308 and S473 (Xu et al., 2005). The mammalian target of rapamycin (mTOR) is an evolutionarily conserved serine–threonine kinase that senses nutrient availability and energy status, promoting protein synthesis, cell growth, and proliferation while inhibiting autophagy. ICV administration of leptin significantly increases the activity of mTOR and its downstream target S6 kinase (S6K), which regulates food intake and energy expenditure. Blocking mTOR with the inhibitor rapamycin largely diminishes the anorectic effects of leptin (Mori et al., 2009). Mice and rats with an S6K null mutation show reduced responsiveness to leptin. On the contrary, rats expressing constitutively active S6K exhibit a more profound appetite-suppressing and weight-reducing response after leptin injection (Blouet et al., 2008). Moreover, ICV injection of leptin fails to induce the phosphorylation of S6K in hypothalamic PI3K-deficient mice, suggesting that PI3K acts as an upstream regulator of mTOR. In addition to its effects on food intake and body weight modulation, mTOR also participates in the regulation of sympathetic nervous activity (SNA) and blood pressure by leptin. Rapamycin can restore the elevated SNA and blood pressure induced by leptin administration (Harlan et al., 2013). Interestingly, chronic activation of mTOR in POMC neurons caused by aging leads to hyperphagic obesity (Yang et al., 2012). This paradoxical phenomenon may be attributed to differences in activation intensity and duration, as well as distinct neuronal cell types and metabolic contexts.

### PI3K-AKT-FOXO1 pathway

Forkhead box protein O1 (FoxO1), targeted by AKT kinase, is a widely expressed transcription factor in the hypothalamus. Its translocation from the cytoplasm to the nucleus leads to increased transcription of Agrp/Npy genes, promoting food intake and body weight gain, while simultaneously decreasing the transcription of *Pomc* gene (Kitamura et al., 2006). Leptin can counteract these effects by inducing the phosphorylation of FoxO1, causing its cleavage from the nucleus. Leptin administration through ICV injection significantly reduces hypothalamic FoxO1 protein expression. Studies show that mice with FoxO1 specifically knocked out in POMC neurons display increased *Pomc* expression, reduced food intake, lower body weight, and enhanced sensitivity to exogenous leptin. Conversely, overexpression of FoxO1 in the hypothalamus impairs leptin signaling and leads to hyperphagic obesity (Kim et al., 2006). Moreover, the balance between STAT3 and FoxO1 further influences the expression of *Pomc* and *Agrp*. STAT3 promotes the activation of *Pomc* and inhibits *Agrp*, whereas FoxO1 stimulates the activation of *Agrp* and suppresses *Pomc*. Mechanically, FoxO1 inhibits leptin-induced transcription of *Pomc* by interfering with the interaction between STAT3 and the transcription factor SP1 (Yang et al., 2009).

### PI3K-PDE3B-cAMP pathway

Phosphodiesterase-3B (PDE3B) is a widely distributed enzyme in the hypothalamus, known for its ability to reduce cyclic adenosine 3′, 5′-monophosphate (cAMP) levels. Accumulating evidence suggests that elevated cAMP levels impair multiple signaling cascades activated by leptin within the hypothalamus. Leptin administration stimulates the activity of PDE3B in the hypothalamus, leading to a reduction in cAMP concentration, which mediates leptin’s anti-obesity effect. However, in conditions such as high-fat diet (HFD) feeding and chronic ICV leptin infusion, the activity of PDE3B in the hypothalamus is impaired. This effect appears to be regulated by the PI3K pathway, as the PI3K inhibitor wortmannin suppresses leptin’s stimulatory effect on PDE3B activity (Sahu and Metlakunta, 2005). Cilostamide, a PDE3B inhibitor, can reverse leptin’s action on food intake and body weight, and it also blocks leptin-stimulated transcription of *Pomc*. Moreover, ICV injection of cilostamide attenuates leptin-induced pSTAT3 activity by blocking the DNA binding of pSTAT3, indicating a crosstalk between the PI3K-PDE3B-cAMP pathway and the JAK2-STAT3 pathway in hypothalamic leptin signaling (Sahu, 2010). A recent study by Sahu et al. revealed that insulin enhances hypothalamic PDE3B activity to reduce food intake and body weight, and this effect can be reversed by cilostamide (Sahu et al., 2017). These findings suggest that PDE3B acts as a novel mediator of insulin signaling in the hypothalamus. Since leptin infusion also stimulates PDE3B activity, further investigations are required to determine whether PDE3B represents an integration point where leptin and insulin signaling pathways converge in the hypothalamus, potentially enhancing their weight-reducing effects.

### SHP2-ERK pathway

ERK (Extracellular Signal-Regulated Kinase) is a vital member of the mitogen-activated protein kinase (MAPK) family, responsible for sensing nutrient conditions and regulating energy balance, cell proliferation, and differentiation. Activation of ERK1/2 by LepRb Tyr985 is facilitated by protein tyrosine phosphatase 2 (SHP2), a Src homology 2-containing tyrosine phosphatase. When leptin is centrally administered, it activates ERK1/2, which contributes to the appetite-suppressing and body weight-reducing effects of leptin (Rahmouni et al., 2009). Mice lacking SHP2 in the brain develop early-onset obesity and leptin resistance. Specifically, deletion of *Shp2* in POMC neurons results in elevated adiposity, reduced energy expenditure, impaired glucose tolerance, and decreased leptin sensitivity (Zhang et al., 2004). Pharmacological inhibitors of ERK1/2 reduce sympathetic nerve activity in brown adipose tissue (BAT), inhibit leptin-induced thermogenesis, and counteract the negative effects of leptin on energy balance. Conversely, SHP2 overexpression in the medial basal hypothalamus (MBH) protects mice from diet-induced obesity by enhancing ERK1/2 activation by leptin (Rahmouni et al., 2009). Recent studies have highlighted the association of ERK1/2 in hypothalamic tanycytes with the transportation of leptin to the hypothalamus. Interestingly, epidermal growth factor (EGF), an activator of ERK signaling, can restore decreased leptin transport and leptin sensitivity in diet-induced obese (DIO) animals (Balland et al., 2014). Given that reduced leptin transport to the hypothalamus contributes to central leptin resistance, it is reasonable to speculate that proper application of EGF may offer a potential adjuvant therapy for obesity and its associated leptin resistance.

## Brain nuclei mediating leptin’s effects on energy balance

Obesity is induced by an imbalance between food intake and energy expenditure, both of which are tightly controlled by the CNS and influenced by peripheral hormones such as leptin (Xu et al., 2011). LepRb exhibits a rich and diverse distribution within the brain, particularly in key hypothalamic nuclei responsible for energy homeostasis regulation, including the ARH, VMH, DMH and LH (Huang et al., 1996; Mercer et al., 1996; Elmquist et al., 1998; Shioda et al., 1998; Funahashi et al., 2003). LepRb’s abundance in these nuclei enables it to engage in complex signaling pathways, influencing neuronal circuits to curb hunger and promote energy expenditure. Beyond the hypothalamic regions, LepRb is also found in extra-hypothalamic areas, such as the midbrain and brainstem (Huang et al., 1996). This extended distribution suggests that leptin’s influence on energy balance is not solely confined to the hypothalamus but extends to more extensive neural networks (Elmquist et al., 1998).

### Role of the ARH in mediating the effects of leptin on energy homeostasis

LepRb exhibits highest density of distribution within the ARH (Elmquist et al., 1998). Although LepRb neurons represent only a minority of ARH cells, their presence is crucial to maintenance of energy balance (Coppari et al., 2005). Within the ARH, two distinct populations of neurons exert opposite functions on energy homeostasis. The first population comprises anorexigenic proopimelanocortin (POMC) neurons, which are activated by leptin (Cowley et al., 2001). The second population consists of orexigenic agouti-related peptide (AgRP)/neuropeptide Y (NPY) neurons that are inhibited by leptin (Pinto et al., 2004). POMC neurons secret alpha Melanocortin stimulating hormone (α-MSH), an agonist of the central melanocortin system that has been extensively studied and recognized as a key neural pathway involved in energy homeostasis regulation. In contrast, AgRP acts as an antagonist of the central melanocortin system (Cone, 2005). Animals deficient for POMC are obese, while ablation of AgRP neurons in adult mice leads to anorexia and leanness (Yaswen et al., 1999; Gropp et al., 2005), highlighting the importance of POMC and AgRP neurons in regulating energy homeostasis.

Early studies originally proposed that POMC neurons play a crucial role in mediating the effects of leptin on body weight. It was observed that leptin administration induces the depolarization of POMC neurons and an increase in the expression of *Pomc* gene (Cowley et al., 2001). POMC-Cre mediated disruption of LepRb results in mild weight gain, while restoration of LepRb expression in POMC neurons has been shown to induce a 15% weight loss in db/db mice (Balthasar et al., 2004; Berglund et al., 2012). However, this notion has been challenged by recent evidence. One perspective is that selectively activating LepRb expressing POMC neurons in mice failed to alter food intake as initially expected (Berglund et al., 2012). More importantly, in prenatal POMC-Cre models, the Cre recombinase was induced in a population of non-POMC neurons (Yu et al., 2022), resulting in unspecific deletion or re-expression of the LepRb encoding gene *Lepr*. Indeed, it has been demonstrated that the POMC-Cre model can activate Cre recombinase in 25% of NPY neurons (Padilla et al., 2010). Additionally, during development, around 87% of LepRb neurons once express *Pomc*, but less than 20% of these cells mature into POMC neurons (Padilla et al., 2012). Given the essential role of leptin signaling pathway in neural development, prenatal deletion of *Lepr* may cause long-term metabolic disturbance in adulthood (Bouret et al., 2004). To avoid these issues, researchers have developed a tamoxifen-inducible POMC-CreER^T2^ model, which allows the targeting of genuine POMC neurons (Caron et al., 2018). Utilizing this model to selectively ablate *Lepr* in mature POMC neurons, researchers found that *Lepr* in POMC neurons is dispensable for the regulation of food intake, energy expenditure and body weight in mice (Caron et al., 2018). This finding was further supported by a study that employed CRISPR-Cas9 mediated viral tool to specifically delete *Lepr* in POMC neurons of mice (Xu et al., 2018).

However, it is worth noting that although leptin signaling in POMC neurons appears not necessary for energy balance under normal chow-fed condition (Caron et al., 2018), its requirement in the context of energy surplus, such as when challenged with a HFD, awaits to be directly investigated. Studies have shown that POMC-specific deletion of *Src1*, a co-activator that enhances pSTAT3 transcriptional activity following leptin treatment, causes hyperphagia and obesity only when mice are fed with a HFD but not chow diet (Yang et al., 2019). Similarly, overexpressing *Grb10* in POMC neurons has no effect on appetite and body weight when mice were fed with chow but alleviates HFD-induced overeating and obesity partly by enhancing hypothalamic leptin sensitivity (Liu et al., 2023). These findings suggest that leptin signaling in POMC neurons is important to ameliorate diet-induced hyperphagia and obesity in the face of overnutrition. Moreover, POMC neurons that express lepRb are important for glucose homeostasis regulation, disrupting *Lepr* in POMC neurons elevated glucose level (Coppari et al., 2005). Conversely, re-expression of *Lepr* selectively in POMC neurons completely normalizes hyperglycemia and improves insulin sensitivity (Morton et al., 2005).

In contrast to POMC neurons, deleting of *Lepr* in AgRP neurons produces hyperphagia and hyperglycemia, resulting in massive weight gain that reaches about 80% of the weight observed in db/db mice (Xu et al., 2018), suggesting AgRP neurons play important roles in mediating leptin’s effects on energy balance. However, recent research has revealed some intriguing insights that provide a more comprehensive understanding. For instance, diphtheria toxin-mediated ablation of AgRP neurons exerts no effects on the appetite-suppressing effects of leptin in ob/ob mice (Zhu et al., 2020), suggesting that the anorexic actions of leptin can still occur even in the absence of AgRP neurons. In addition, while chronic inhibition of AGRP neurons fails to alter food intake and body weight in ob/ob mice, chronic inactivation of all GABAergic neurons in the ARH largely ameliorates hyperphagia, hyperglycemia and obesity in mice lacking leptin (Zhu et al., 2020). Taken together, these observations suggest that while AgRP neurons play important roles in mediating the effects of leptin on energy balance, they may not be the sole mediators. Other neuronal populations, including non-AgRP GABAergic neurons, may also contribute to the anorexigenic and weight-reducing actions of leptin. For instance, NPY released by AgRP-negative NPY neurons located in the ARH exerts an inhibitory influence on POMC neurons. This inhibition reduces the responsiveness of POMC neurons to leptin, ultimately contributing to overeating and the development of obesity (Qi et al., 2023).

### Role of the VMH in mediating the effects of leptin on energy homeostasis

The VMH has long been recognized as a key site that regulates energy and glucose homeostasis, and neurons express steroidogenic factor 1 (SF-1) are pivotal in this process (Krause and Ingraham, 2017). The SF-1 neurons release brain-derived neurotrophic factor (BDNF), selective deletion of BDNF in the VMH results in hyperphagia and obesity in mice (Liao et al., 2012). Moreover, the VMH contains a large population of LepRb neurons, most of which express SF-1 and the neuropeptide pituitary adenyl cyclase activating protein (PACAP; Hawke et al., 2009; Kim et al., 2011). Deletion of *Lepr* in SF-1/PACAP neurons did not produce substantial effects on body weight under normal chow-fed conditions but led to increased fat mass in mice. However, when these mice were challenged with a HFD, the absence of LepRb in SF1 neurons accelerated the development of obesity primarily by diminishing energy expenditure (Dhillon et al., 2006). Remarkably, PACAP has been identified as a crucial mediator of the central effects of leptin on energy balance. Administration of PACAP antagonists significantly attenuated the hyperthermic response induced by leptin (Hawke et al., 2009), indicating the importance of PACAP in mediating leptin’s effects on body temperature regulation.

Notably, studies have shown that systemic injection of leptin triggered an increase in plasma catecholamine concentrations, and such effect was blocked in rats with VMH lesions (Satoh et al., 1999). This suggests that the VMH plays a role in the sympathetic activation elicited by leptin. Additionally, microinjection of leptin into the VMH has been shown to not only increase blood pressure and renal sympathetic activity, but also enhance glucose uptake in various tissues, including BAT, heart, skeletal muscles, and spleen (Minokoshi et al., 1999). It is worth mentioning that when the interscapular BAT was subjected to sympathetic denervation, the augmented glucose uptake in response to leptin administration was completely abolished (Haque et al., 1999), highlighting the involvement of sympathetic innervation in mediating the enhanced glucose uptake induced by leptin. Consistently, deprivation of the small GTPase Rap1 or Gsα in SF-1 neurons ameliorates diet-induced leptin resistance and improves glucose homeostasis (Berger et al., 2016; Kaneko et al., 2021). By contrast, disruption of LIM domain only 4 (LMO4) in the VMH attenuates thermogenesis and leads to obesity. Particularly, LMO4 knockout mice exhibit impaired BAT thermogenesis in response to leptin (Zhou et al., 2012), emphasizing the permissive role of LMO4 signaling in the VMH for leptin-mediated energy expenditure. Carnitine palmitoyltransferase 1C (CPT1C) deficiency in the VMH promotes leptin resistance and impairs BAT thermogenesis (Rodríguez-Rodríguez et al., 2019), underscoring the importance of CPT1C in counteracting obesity. Conversely, deletion of the transcription factor FoxO1 in SF-1 neurons increases BAT thermogenesis and energy expenditure, resulting in a lean phenotype in mice. Additionally, mice lacking FoxO1 show improved leptin sensitivity when exposed to a HFD (Kakar, 2015). These findings collectively demonstrate that leptin signaling within the VMH counteracts diet-induced obesity by promoting thermogenesis and enhancing energy expenditure.

### Role of the DMH in mediating the effects of leptin on energy homeostasis

The DMH has emerged as a key regulator of feeding, energy expenditure and body weight. Lesions in the DMH reduce food intake, prevent diet-induced obesity and disrupt circadian rhythms (Seoane-Collazo et al., 2015). Various neuropeptides and receptors, such as neuropeptide Y (NPY), prolactin-releasing peptide (PrRP) and lepRb, are expressed within the DMH (Chao et al., 2011; Dodd et al., 2014; Faber et al., 2021). Intra-DMH injection of leptin elicits increases in body temperature, heart rate and blood pressure, not only in lean mice but also in obese mice (Marsh et al., 2003; Enriori et al., 2011), suggesting the DMH is sensitive to leptin even in obese conditions. Activation of LepRb neurons within the DMH using chemogenetics enhances BAT thermogenesis, energy expenditure and body temperature without altering food intake, leading to weight loss (Rezai-Zadeh et al., 2014). Conversely, blocking leptin action in the DMH through the use of antibodies, LepRb antagonists or ablation of LepRb expressing neurons, decreases heart rate and blood pressure (Simonds et al., 2014). Interestingly, while diet-induced obese mice exhibit elevated heart rate and blood pressure, mice lacking leptin or leptin receptors maintain normal blood pressure despite severe obesity. Consistently, re-expression of *Lepr* in the DMH of db/db mice raises blood pressure (Simonds et al., 2014). Notably, disruption of *Lepr* selectively in RrRP neurons of the DMH impairs BAT thermogenesis and causes obesity in mice (Dodd et al., 2014). Loss of Gsα in the DMH of mice impairs leptin signaling, increases food intake and decreases energy expenditure, suggesting that the anti-obesity effects of leptin are at least partially mediated by Gsα (Chen et al., 2019). LepRb neurons play a crucial role in the comprehensive circadian regulation of feeding and energy balance. Silencing LepRb neurons specifically in the DMH leads to a phase advance in diurnal rhythms of feeding and metabolism, causing them to align with the light cycle (Faber et al., 2021). This silencing also eliminates the typical increase in locomotor activity during the dark cycle observed in nocturnal rodents, ultimately resulting in obesity (Faber et al., 2021). Furthermore, DMH LepRb neurons that project to the suprachiasmatic nucleus exhibit calcium activity prior to an anticipated meal. These neurons serve as a crucial link between the metabolic and circadian systems, thus facilitating mealtime anticipation (Tang et al., 2023).

The sympathetic nervous system (SNS) mediates the cardiovascular and thermogenic effects of leptin within the DMH. Intra-DMH leptin infusion or chemogenetic activation of DMH LepRb neurons in rodents induces an increase in BAT temperature (Zhang et al., 2011). However, pretreatment with a β3-antagonist blocks this increase, emphasizing the involvement of the SNS in leptin-dependent BAT thermogenesis (Zhang et al., 2011). LepRb neurons in the DMH project to various areas, including the PVH, periaqueductal gray (PAG), and raphe pallidus (RPa), which are known to regulate the SNS’s control over body temperature and cardiovascular system (Zhang et al., 2011). The specific role and identity of these projections are yet to be fully understood. Moreover, it remains unclear whether leptin exerts its different effects, such as thermogenesis, blood pressure, and heart rate, through distinct neuronal populations. While leptin receptors are expressed in both GABAergic and glutamatergic neuronal populations (Rusyniak et al., 2008), the precise neuronal subtypes that mediate the effects of leptin on thermogenesis, heart rate, and blood pressure are not completely known. Inhibiting glutamate release from LepRb neurons decreases energy expenditure and body temperature in mice, suggesting that glutamate release plays a role in mediating leptin’s action on energy expenditure (Xu et al., 2013). Interestingly, activating neurons expressing the bombesin-like receptor 3 (Brs3) in the DMH of mice increases body temperature, BAT temperature, energy expenditure, heart rate, and blood pressure without affecting food intake or physical activity. Additionally, optogenetic stimulation of DMH Brs3 neuronal terminals in the RPa raises body temperature (Piñol et al., 2018). Furthermore, a significant subset of Brs3 neurons in the DMH co-express LepRb, suggesting leptin might play a crucial role in mediating the effects of Brs3 neurons on energy homeostasis. Recently, it has been shown that α3-GABA_A_ receptor expressing neurons within the DMH act downstream of AgRP neurons and mediate the effects of leptin on food intake and energy balance in mice (Han et al., 2023). A more comprehensive understanding of the neuronal networks and circuits that respond to leptin will facilitate the development of novel strategies to alleviate leptin resistance and counteract obesity.

### Role of the PVH in mediating the effects of leptin on energy homeostasis

The PVH plays a crucial role in the regulation of appetite and endocrine function (Sutton et al., 2016). It comprises distinct subpopulations of neurons, including oxytocin (OXT), vasopressin (AVP), corticotropin-releasing hormone (CRH), thyrotropin-releasing hormone (TRH) and BDNF expressing neurons, which collectively govern various physiological processes (Qin et al., 2018). Notably, the specific involvement of LepRb signaling in the PVH in mediating leptin’s effects has been the subject of controversy. On one hand, leptin receptors are present in the PVH, and *in vitro* slice recordings have shown that a majority of PVH neurons are directly depolarized by leptin, indicating that they are responsive to leptin signaling (Powis et al., 1998). Notably, leptin activates OXT neurons in the PVH, leading to the inhibition of food intake, suggesting that OXT neurons partially mediate the anorexic effect of leptin (Hakansson and Meister, 1998). Furthermore, AVP neurons in the PVH co-express leptin receptors, and central administration of leptin enhances AVP mRNA expression in the PVH and plasma AVP levels, indicating that AVP neurons could be potential targets of leptin (Yamamoto et al., 1999). On the other hand, it is important to note that the PVH receives inputs from other essential hypothalamic nuclei and the brainstem, and transmits signals to regulate critical physiological processes, including food intake, energy expenditure, and autonomic functions. Moreover, the PVH also interacts with the endocrine axes, affecting hormonal secretions and further influencing metabolism and energy utilization (Liu et al., 2010). Indeed, most PVH neurons express melanocortin receptors and neuropeptide Y (NPY) receptor subtypes, and receive dense projections from the ARH and VMH (Campbell et al., 2001; Wang et al., 2020; Bouret, 2022). It has been shown that leptin signaling in hypothalamic POMC and AgRP neurons modulates the sympathetic innervation of subcutaneous white and brown adipose tissue, these effects are mediated by BDNF in the PVH (Wang et al., 2020). Moreover, the PVH is innervated by LepRb-expressing neurons in the DMH (Myers et al., 2009). Hence, leptin action in the PVH involves inputs from leptin-responsive neurons originating from other brain regions.

### Role of the LH in mediating the effects of leptin on energy homeostasis

The involvement of leptin signaling within the LH in regulating energy homeostasis has been well-documented (Leinninger, 2011). One compelling piece of evidence supporting this is the observation that intra-LH administration of leptin in ob/ob mice, which lack functional leptin, leads to a reduction in appetite and body weight (Leinninger et al., 2009). Conversely, selectively knocking down *Lepr* specifically in the LH induces hyperphagia and obesity (Davis et al., 2011). However, a recent study showed that manipulating the activity of LepRb neurons in the LH has no effect on food intake but rather influences appetitive learning (Siemian et al., 2021). Subsequent investigations revealed the presence of two distinct populations of LepRb neurons in the LH, each playing a specific role during different phases of feeding behaviors. Optogenetic stimulation of LepRb neurons in the LH exclusively during the seeking phase led to an enhancement in seeking behavior in mice. Similarly, optogenetic stimulation of LH LepRb neurons specifically during the consummatory phase resulted in increased food consumption. Intriguingly, when LH LepRb neurons were stimulated at both the seeking and consummatory phases, no discernible effects on seeking or consummatory behaviors were observed (Lee et al., 2023). Thus, these studies provide valuable insights into the intricate functions of two distinct neuronal populations expressing LepR in the LH.

Leptin exerts its influence on multiple neural populations in the LH, including neurotensin (NTS)-, orexin-, and melanin-concentrating hormone (MCH)-expressing neurons (Leinninger et al., 2011). Notably, a significant proportion of neurotensin neurons in the LH express LepRb and can be directly activated by leptin (Leinninger et al., 2011). In line with this, deletion of *Lepr* in LH neurotensin neurons reproduces the hyperphagic and obese phenotype observed in mice with LepRb deficiency in the LH (Leinninger et al., 2011). These findings indicate that LepRb-expressing neurotensin neurons play a crucial role in controlling energy balance. With respect to orexin neurons in the LH, it is important to note that recent studies suggest they belong to a distinct neural population from LepRb-expressing neurons (Leinninger et al., 2009). Therefore, it is likely that orexin neurons are indirectly modulated by leptin. Leptin injection suppresses the firing of orexin neurons and inhibits the release of orexin, leading to a reduction in food intake (Tritos et al., 2001). However, since orexin neurons and LepRb neurons in the LH are separate populations, it is believed that orexin neurons mediate the actions of leptin indirectly, likely via receiving inputs from local neurotensin neurons and other leptin-responsive neurons. Similar to orexin neurons, MCH neurons in the LH do not express LepRb. Consequently, the impact of leptin on the suppression of MCH mRNA and neuronal activity are believed to be indirect (Sahu, 1998a,b; Tritos et al., 2001), possibly through innervations by a number of other neuronal populations.

Interestingly, emerging research has revealed that early-life trauma can have profound effects on leptin signaling within the LH, leading to long-term alterations in behavior and metabolism. Specifically, it has been demonstrated that early-life trauma in mice downregulates the expression of *Lepr* mRNA and enhances the activity of LepRb-expressing neurons in the LH (Shin et al., 2023). These changes contribute to increased binge-eating and obesity later in life. The mechanisms underlying this effect involve proenkephalin-expressing neurons located in the ventrolateral periaqueductal gray (vlPAG; Shin et al., 2023). Within the LH, LepRb neurons play a crucial role in coordinating competing physiological needs. They effectively suppress feeding and drinking behaviors and promote social interaction, even in the presence of hunger or thirst (Petzold et al., 2023). In contrast, neurotensin-expressing neurons in the LH exhibit a distinct preference for encoding the need for water. These neurons promote water-seeking behaviors while concurrently downplaying social needs (Petzold et al., 2023). Thus, LepRb- and neurotensin-expressing neurons in the LH work in tandem, acting complementarily to enable the flexible fulfillment of multiple essential needs.

### Role of the POA in mediating the effects of leptin on energy homeostasis

LepRb neurons not only participate in the regulation of energy balance but are also integral components of a thermoregulatory circuit that connects the preoptic area (POA), a well-established brain region responsible for body temperature regulation, with the sympathetic control of BAT (Rothhaas and Chung, 2021). LepRb neurons in the POA are exclusively glutamatergic and are specifically activated by warm temperatures (Yu et al., 2016). Activation of these neurons through chemogenetic techniques robustly inhibits energy expenditure and leads to a reduction in BAT temperature (Yu et al., 2016). Leptin signaling within the POA serves to modulate weight gain in states of energy surplus and prevent the decrease in energy expenditure that typically occurs during fasting (Yu et al., 2016, 2018a). This suggests that LepRb neurons in the POA play a vital role in integrating leptin signaling with energy adaptations. Notably, the POA sends dense projections to the DMH and the RPa (Nakamura et al., 2002; Zhao et al., 2017). A study utilizing rabies virus based retrograde tracing from the BAT has identified the POA as one of the upstream regulators involved in this circuit, with LepRb neurons playing a key role (Zhang et al., 2011).

Furthermore, activation of LepRb neurons within the POA has been shown to suppress food intake in response to ambient temperatures, leading to weight loss in mice (Kaiyala et al., 2015; Yu et al., 2018b). Intriguingly, leptin-deficient mice lose the ability to reduce food intake in warm temperatures, emphasizing the crucial role of leptin in this thermoregulatory mechanism (Kaiyala et al., 2015). Another recent study has shed light on the distinct populations of glutamatergic neurons in the POA that are involved in mediating feeding behavior. Specifically, two neighboring but functionally separate neuronal populations have been identified. The first group consists of galanin receptor expressing POA neurons that project to the ARH and are activated by low temperatures to promote food intake. In contrast, the second group comprises apelin receptor containing POA neurons that project to the PVH and primarily respond to high temperatures, resulting in the suppression of feeding in mice (Qian et al., 2022). However, additional investigation is required to determine whether LepRb expression is present in these specific neuronal populations within the POA.

### Role of the VTA in mediating the effects of leptin on energy homeostasis

Leptin signaling within the ventral tegmental area (VTA) plays a crucial role in the regulation of energy homeostasis, and its dysfunction has been implicated in obesity and metabolic disorders (Figlewicz et al., 2006). Dopamine (DA) neurons within the VTA project extensively to the nucleus accumbens (NAc), where they modulate motivation and reward-related behaviors (Pierce and Kumaresan, 2006). It is noteworthy that a significant portion of LepRb-expressing neurons in the VTA are dopaminergic. Leptin administration has been shown to affect DA synthesis in the VTA and DA reuptake in the NAc (Fulton et al., 2006; Opland et al., 2010). Injection of leptin directly into the VTA leads to a reduction in food intake, while RNAi-mediated knockdown of *Lepr* specifically in the VTA promotes feeding behavior (Hommel et al., 2006). These findings highlight the importance of leptin signaling within the VTA in the regulation of food intake. Interestingly, it has been observed that ablating *Lepr* in DA neurons does not have a significant impact on motivation (Fulton et al., 2000). However, it does result in increased anxiety-like behavior (Liu et al., 2011). These findings suggest that non-dopaminergic LepRb-expressing neurons in the VTA may play a crucial role in mediating the effects of leptin on the dopamine system and associated behaviors.

The VTA is densely innervated by LepRb neurons in the LH, suggesting a potential role for LH LepRb neurons in the control of mesolimbic DA function (Leinninger et al., 2009, 2011). This notion is supported by research showing that intra-LH leptin injection increases the expression of tyrosine hydroxylase, an enzyme involved in DA production, in the VTA (Leinninger et al., 2009). Furthermore, LH LepRb neurons project to the VTA and have been found to modulate appetitive behaviors (Leinninger et al., 2009). Leptin inhibits LepRb neurons in the LH, which disinhibits GABAergic neurons in the VTA, resulting in subsequent suppression of DA neurons (Leinninger et al., 2009). Activation of LH LepRb neurons enhances motivation, while their inhibition decreases motivation (Fulton et al., 2000; Omrani et al., 2021). These effects are likely mediated by non-DA neurons in the VTA, indicating the involvement of diverse neuronal populations in the LH-VTA circuit. Moreover, glutamatergic neurons in the LH also provide inputs to the VTA (Rossi et al., 2021). Given the existence of multiple peptidergic projections from the LH to the VTA, it is important for future studies to investigate how the activation of different LH inputs coordinates the activity of VTA DA and non-DA neurons and subsequently influences their output. Specifically, the specific impact of various peptidergic LH inputs on dopaminergic output in target regions remains unclear. It is essential to explore circumstances under which multiple peptidergic LH inputs to the VTA are simultaneously activated and understand the coordinated effects they exert on dopaminergic signaling in relevant brain regions.

### Role of the NTS in mediating the effects of leptin on energy homeostasis

LepRb is expressed in the nucleus tractus solitarius (NTS) and area postrema (AP). In the NTS, LepRb neurons consist of distinct neural populations expressing proopiomelanocortin, proglucagon, and cholecystokinin (CCK; Garfield et al., 2012). Studies have demonstrated the crucial role of LepRb signaling in these brain regions for appetite control and metabolism. Knockdown of Lepr in the NTS and AP leads to hyperphagia and obesity in rats. Furthermore, impairment of LepRb signaling in the NTS and AP blunts the response to gastrointestinal satiation signaling, particularly CCK (Hayes et al., 2010). Conversely, chemogenetic activation of LepRb neurons in the NTS suppresses food intake, and this effect remains unaffected by ablating glucagon-like peptide-1 (GLP-1) in NTS LepRb neurons (Cheng et al., 2020). Moreover, the anorexigenic effects of leptin seem to involve BDNF/TrkB signaling in the NTS. Pretreating the NTS with a TrkB antagonist diminishes the appetite-suppressing effects of leptin, suggesting the involvement of BDNF and its receptor TrkB in mediating these effects (Spaeth et al., 2012). Unlike ARH POMC neurons, which are responsive to leptin, NTS POMC neurons do not show altered c-fos expression upon leptin administration, suggesting that POMC neurons in the NTS are unresponsive to leptin (Huo et al., 2006). Notably, activating NTS LepRb neurons in obese mice leads to increased ventilation, indicating a potential link between NTS LepRb and breathing regulation (Do et al., 2020).

### Role of non-neurons in mediating the effects of leptin on energy homeostasis

Recent evidence has shed light on the interplay between neurons and non-neuronal cells within the brain in regulating leptin response and energy balance. Beyond neurons, various non-neuronal cell types in the CNS actively participate in the complex regulation of energy balance. For instance, glial cells, particularly astrocytes and microglia, have emerged as essential players in leptin signaling. These glial cells express functional leptin receptors and respond to leptin by modulating their metabolic and inflammatory activities (Hosoi et al., 2000; Pan et al., 2008; Nampoothiri et al., 2022). Leptin treatment induces interleukin-1β and tumor necrosis factor-α expression in hypothalamic microglia, whereas the disruption of leptin signaling in ob/ob or db/db mice leads to alterations in genes associated with microglial functions (Gao et al., 2014). By influencing the neuroinflammatory environment and providing metabolic support to neurons, glial cells contribute significantly to the regulation of energy homeostasis. For instance, deletion of LepRb within microglia diminishes the number of POMC neurons in the ARH, ultimately accelerating in the onset of obesity (Gao et al., 2018). Despite the absence of colocalization between BDNF and LepRb, disrupting BDNF/TrkB.T1 signaling in the VMH astrocytes diminishes the impact of leptin on energy homeostasis, suggesting astrocyte BDNF signaling plays a permissive role in mediating leptin action (Ameroso et al., 2022). Likewise, dysfunction of the primary cilia in the CNS has been associated with hyperphagia, hypothermia, leptin resistance and obesity (Sun et al., 2021). Additionally, ablation of tanycytes in mice blunts the transport of leptin into the brain and results in overeating, insulin resistance and obesity (Balland et al., 2014). In mice lacking LepRb or those with diet-induced obesity, leptin, when taken up by tanycytes, tends to accumulate in the median eminence, failing to reach the ARH. Conversely, when ERK signaling in tanycytes is activated, it reinstates leptin transport, thus enhancing leptin sensitivity (Balland et al., 2014). Furthermore, endothelial cells within the brain’s vasculature also play a vital role in the transport of leptin across the blood–brain barrier, facilitating leptin’s access to specific brain regions involved in appetite control. Understanding the interplay between neurons and non-neuronal cells in mediating leptin’s effects on energy balance is crucial for gaining comprehensive insights into the complex neural networks governing metabolic regulation and may offer potential targets for therapeutic interventions in obesity and related metabolic disorders.

## Conclusion

**Figure 1:**
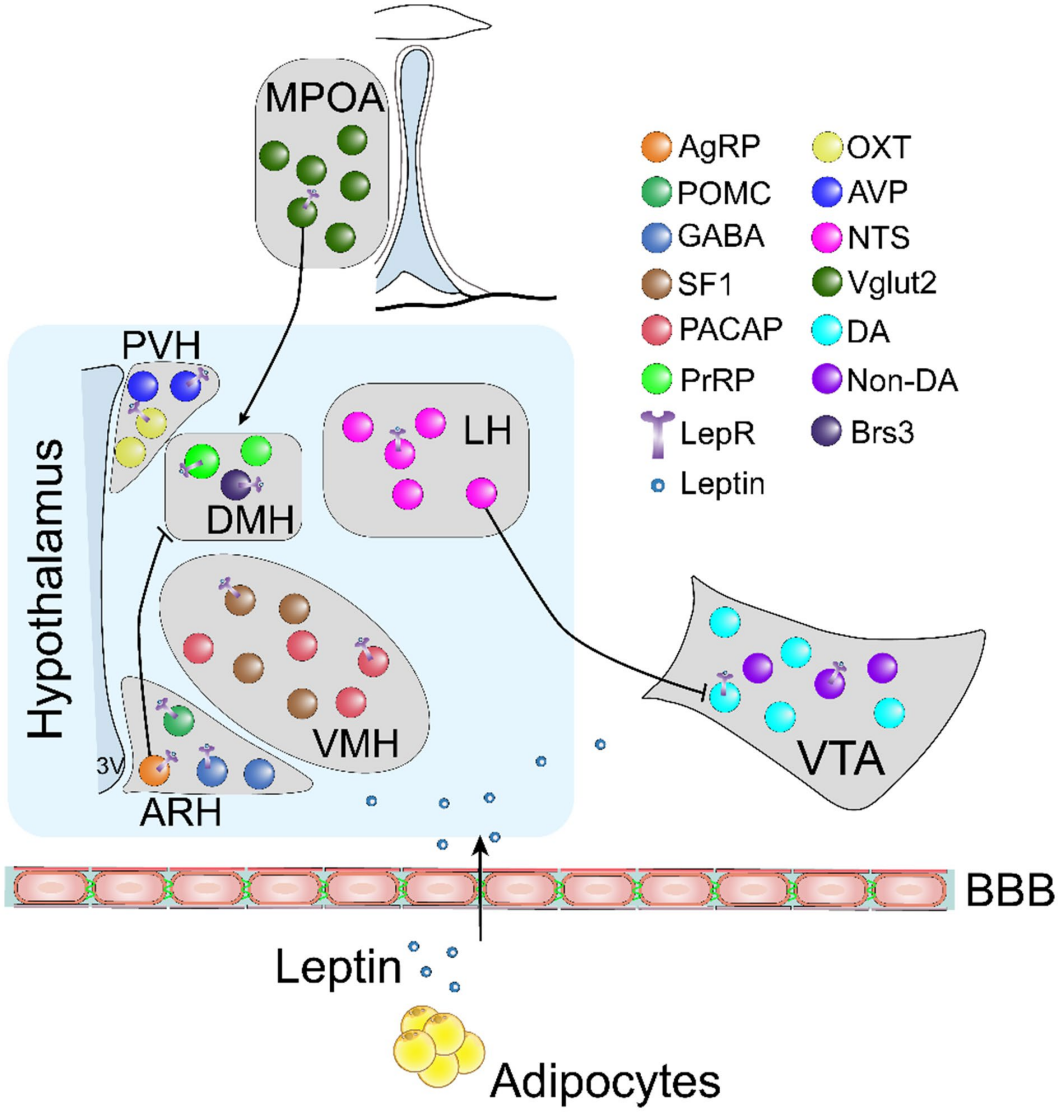
An illustrative schematic diagram showing key neural subtypes involved in mediating the effects of leptin on food intake and energy expenditure. BBB, blood brain barrier; AgRP, agouti-related peptide; POMC, proopiomelanocortin; GABA, gamma aminobutyric acid; SF-1, steroidogenic factor 1; PACAP, pituitary adenyl cyclase activating protein; PrRP, prolactin-releasing peptide; OXT, oxytocin; AVP, vasopressin; NTS, neurotensin; Vglut2, vesicular glutamate transporter 2; DA, dopamine; Brs3, bombesin-like receptor 3; LepR, leptin receptor.

This review provides an overview of the key signaling pathways and neural populations responsible for mediating the effects of leptin on energy balance (Figure 1). Significant strides have been made in understanding the functional role of leptin in regulating energy metabolism. However, there is a notable absence of therapeutic strategies targeting leptin signaling pathways, underscoring the need to deepen our understanding of leptin signaling and the neuronal networks involved in its function. A crucial obstacle to achieving the full potential of leptin is the development of leptin resistance. As such, the identification of novel molecules capable of enhancing leptin sensitivity is a critical step in the development of new therapeutic approaches to address obesity. In addition, while leptin receptors are primarily expressed in the hypothalamus, their expression extends to other brain regions that play crucial roles in energy homeostasis. One important area that requires further exploration is the interactions between distinct subsets of LepRb-expressing neurons and their downstream targets. Understanding these intricate neural networks is essential for formulating targeted strategies that leverage leptin to combat obesity effectively. Moreover, it is worth noting that LepRb is expressed in astrocytes and microglia, emphasizing the importance of investigating the function of leptin on these non-neuronal cells and their potential impact on neurons in the context of energy balance. Such research will provide valuable insights into the interplay between different cell types and their contributions to the overall effects of leptin.

## Author contributions

ZL, TX, and HL conceptualized and wrote the manuscript. All authors contributed to the article and approved the submitted version.
